# Impact of an Educational Hands-on Project on the Antimicrobial, Antitumor and Anti-Inflammatory Properties of Plants on Portuguese Students’ Awareness, Knowledge, and Competences

**DOI:** 10.3390/ijerph120302437

**Published:** 2015-02-23

**Authors:** Maria-Manuel Azevedo, Céline Pinheiro, Alberto C.P. Dias, Filipa Pinto-Ribeiro, Fátima Baltazar

**Affiliations:** 1 School D. Maria II, Rua da Alegria, 4760-067 Vila Nova de Famalicão, Portugal; 2 Department of Microbiology, Faculty of Medicine, University of Porto, 4200-319 Porto, Portugal; 3 Life and Health Sciences Research Institute (ICVS), School of Health Sciences, University of Minho, 4710-057 Braga, Portugal; E-Mails: cpinheiro@ecsaude.uminho.pt (C.P.); filiparibeiro@ecsaude.uminho.pt (F.R.); 4 ICVS/3B’s-PT Government Associate Laboratory, 4710-057 Braga/Guimarães, Portugal; 5 Barretos School of Health Sciences, Dr. Paulo Prata—FACISB, 14784-400 Barretos, São Paulo, Brazil; 6 Molecular Oncology Research Center, Barretos Cancer Hospital, Pio XII Foundation, 14784-400 Barretos, Brazil; 7 Center for the Research and Technology of Agro-Environmental and Biological Sciences (CITAB), Department of Biology, University of Minho, Campus de Gualtar, 4710-057 Braga, Portugal; E-Mail: acpdias@bio.uminho.pt

**Keywords:** innovation in education, experimental/field work, autochthonous plants, pharmacological properties of plant extracts, experimental models of disease

## Abstract

Promoting environmental and health education is crucial to allow students to make conscious decisions based on scientific criteria. The study is based on the outcomes of an Educational Project implemented with Portuguese students and consisted of several activities, exploring pre-existent Scientific Gardens at the School, aiming to investigate the antibacterial, antitumor and anti-inflammatory properties of plant extracts, with posterior incorporation in soaps and creams. A logo and a webpage were also created. The effectiveness of the project was assessed via the application of a questionnaire (pre- and post-test) and observations of the participants in terms of engagement and interaction with all individuals involved in the project. This project increased the knowledge about autochthonous plants and the potential medical properties of the corresponding plant extracts and increased the awareness about the correct design of scientific experiments and the importance of the use of experimental models of disease. The students regarded their experiences as exciting and valuable and believed that the project helped to improve their understanding and increase their interest in these subjects and in science in general. This study emphasizes the importance of raising students’ awareness on the valorization of autochthonous plants and exploitation of their medicinal properties.

## 1. Introduction

The importance of environmental education has increased in the same proportion that public awareness increases regarding the severity of plant loss, such as autochthonous plants. In Portugal, the expansion of exotic plants constitutes a threat for autochthonous plants and has become a serious environmental problem [1]. Sometimes, exotic plants develop new morphologies and reproductive behaviors to compete successfully in their new habitats [2] and they are able to spread over enormous regions and considerable distances from the parent plants [3]. Controlling this type of plants is extremely difficult and costly, so upstream measures, such as avoiding their initial introduction, are needed [4].

To increase awareness of this issue and recognize the value of autochthonous plants, we have developed several environmental education activities with 8th grade students, including the didactic exploitation of the three scientific gardens at the school. Plants are a viable and unlimited source of bioactive molecules, and throughout history, they have been an important source of medicines with antibacterial, antitumor and anti-inflammatory properties. According the World Health Organization, medicinal plants are the best source from which to obtain a variety of drugs [5].

An increasing number of microorganisms that cause disease are becoming resistant to the antimicrobial agents commonly used in the clinical setting, so finding new alternatives is a priority [6], for example, by further investigating the antimicrobial activity of plants. There are numerous plants with antimicrobial activity [7,8,9,10,11]. Recently, the antibacterial potential of phenolic extracts recovered from plants collected in the northeast Portugal against bacteria found in skin infections was demonstrated [10]. According to this work, extracts of *Cistus ladanifer*, *Castanea sativa*, *Filipendula ulmaria* and *Rosa micrantha* revealed promising antibacterial effects against *Klebsiella pneumonia*, *Staphylococcus epidermidis* and *Staphylococcus aureus*. Other studies showed the antimicrobial activity of plant extracts of *Cinnamomum verum* and *Syzygium aromaticum* against several pathogenic strains, with the former inhibiting the growth of *Bacillus subtilis* and *Candida albicans* and the latter the growth of *Bacillus subtilis*, *C. albicans* and *Pseudomonas aeruginosa* [12]. In addition, extracts of *Artemisia annua* were shown to have antimicrobial activity against *Escherichia coli* [13].

Cancer is one of the most devastating diseases in humans, and interestingly, many anti-cancer agents were developed based on natural products extracted from plants. Experiments with human breast adenocarcinoma and human lung carcinoma cell lines demonstrated that methanol extracts of *A. annua* exhibited significant anticancer activity [13]. Sharma and co-workers (2011) [14] evaluated the activity of the methanolic extract of *Glochidion zeylanicum* using the human cancer cell lines HepG2 (liver cancer), HT-29 (colon cancer) and PC-3 (prostate cancer). The results demonstrated that this extract has a potential cytotoxicity against HepG2, HT-29 and PC-3 cell lines but not against healthy cells, although the effect was more pronounced in the PC-3 cell lines. Additionally, extracts prepared from *Ononis hirta* and *Inula viscosa* were effective against MCF-7 cells (breast epithelial adenocarcinoma) by inducing apoptosis [15]. Yu and collaborators (2013) [16] showed the antitumor activity of *Rauwolfia vomitoria* extract against three ovarian cancer cell lines [17]. This extract inhibited tumor growth in a mouse model with intraperitoneal metastasis and massive ascites formation, either alone or in combination with other therapies. A previous work reported that *R. vomitoria* extracts caused DNA damage and cell cycle inhibition in prostate cancer cells [18].

In many countries, plants are also widely used to treat inflammatory conditions, in particular skin inflammation. The phenolic compounds obtained from plant extracts have considerable anti-inflammatory properties. A study conducted by Falcão and co-workers [19] reported a list of seventy-five plant species with anti-inflammatory activity. The leaves of *Bouchea fluminensis* contain iridoid and steroid glycosides that have anti-inflammatory properties. The extract of *Hyptis pectinata* leaves similarly exhibited a significant antiedematogenic activity [19]. Another work studied the effect of *Pterodon pubescens* seed extract in an arthritis animal model for preventive and therapeutic antiarthritis treatment [16]. These authors demonstrated that preventive treatment significantly reduced the arthritic index and arthritis incidence [16]. Franzotti and co-workers [20] discovered the anti-inflammatory properties of *Sida cordifolia* plant extract, and Gupta and co-workers [21] discovered that plant extracts of *Bauhinia racemosa*, a small tree widely distributed throughout India, Ceylon, China and Timor, also possess potent anti-inflammatory activity [21].

Hands-on activities are great student motivators, not only by improving the quality of science education but also by awakening environmental and ethical awareness. Direct contact with living organisms provides information and experiences that are not accessible by reading, viewing pictures or examining models [22]. Thus, we developed a hands-on activity with basic-school students. We selected six autochthonous plants from Portugal, harvested from the school’s scientific gardens—*Cistus populifolius*, *Crataegus monogyna*, *Erica australis*, *Helichrysum stoechas*, *Lavandula pedunculata* and *Rosa canina*—and we prepared plant extracts and assessed their antimicrobial, anti-inflammatory and antitumor properties. The experimental activities were performed with Portuguese 8th grade students from D. Maria II School, V.N. Famalicão.

The interest of students in subjects such as plant properties can increase significantly if teachers and researchers’ are involved, when appropriate methodologies are used. According to Azevedo *et al.*, laboratory work seems to be valuable in Science Education [23] and another recent study describes the educational benefits of incorporating hands-on activities in science education programs [24]. In addition, the study of Azevedo *et al.* [25] also supports the use of experimental activities in an informal environment as learning tools to increase scientific knowledge.

**Figure 1:**
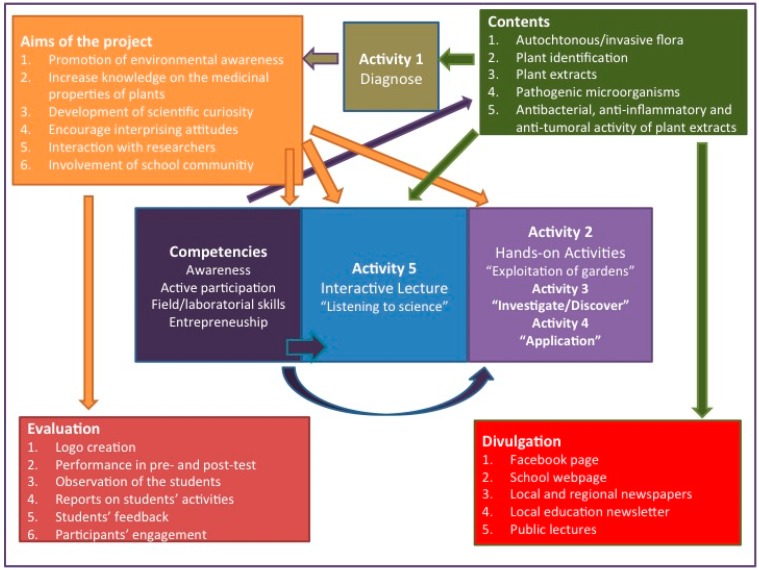
Diagram illustrating the rationale of the project.

The activities developed in this study with the 8th grade students included: (0) Creation of a logo and a webpage; (1) assessment of previous knowledge on the subjects (pre-test); (2) maintenance of three scientific gardens at the school and harvesting of selected plant samples; (3) preparation of plant extracts and evaluation of their antimicrobial, antitumor and anti-inflammatory properties; (4) incorporation of the extracts in soaps and creams; (5) organization of scientific lectures and discussions; (6) reporting of the results in the community, with development of communication strategies; and (7) evaluation of the activities developed (post-test). An overview of the project is available in Figure 1.

The objectives of this study were: (a) to promote environmental awareness through the knowledge of our autochthonous flora by involving the students in field/experimental work; (b) to increase students’ knowledge on the antibacterial, antitumor and anti- inflammatory properties of autochthonous plants; (c) to increase the students’ scientific curiosity and foster critical thinking and attitudes; (d) to encourage enterprising attitudes in students; (e) to put students in close contact with university laboratories and researchers; (f) to instigate the involvement of the school community in the development of scientific activities. To validate all of the activities and assess student learning, students were asked to answer a questionnaire on various topics before and after the laboratory/field activities (pre-test and post-test).

## 2. Experimental Section

### 2.1. Research Questions

The specific research questions addressed by this project were: to what extent: (a) are students familiar with the term autochthonous plants and can identify some Portuguese autochthonous plants; (b) are students familiar with the term plant extracts and how these can be prepared; (c) are students familiar with the antimicrobial, antitumor and anti-inflammatory properties of plants; (d) can students classify microorganisms into their main categories, and identify some pathogenic ones; (e) are students familiar with the importance of the correct design of scientific experiments and the value of experimental models; students know how new medicines are discovered; (f) these activities motivate students.

### 2.2. Participants

The activities of this study were developed between March and June 2014. This research project involved two schools (D. Maria II (*n* = 19) and Arnoso S. Maria (*n* = 19), [control group]), both belonging to School Cluster D. Maria II. V.N. Famalicão, Braga district (Table 1). For this work we used a convenience sample. The participants (D. Maria II: females (*n* = 6) and males (*n* = 13) aged between 13 and 15 years old; Arnoso Sta. Maria: females (*n* = 8) and males (*n* = 11) aged between 12 and 16 years old) attended the 8th grade (Table 1). This study was approved by the School Board of D. Maria II, V. N. Famalicão, after a hearing by the Pedagogic Council because there is no ethics committee at the school. Student participation was anonymous and voluntary. Informed consent was obtained verbally from the students’ guardians on behalf of the students enrolled in our study. This consent was obtained during a regular meeting, in which the director of the class explained the aims of the project and requested authorization from the parents for their children to participate. Verbal consent was agreed upon by the School Board, class director and the students’ guardians. Written informed consent was obtained from the students’ guardians for the pictures shown in the manuscript. All the other data used in this study was anonymized.

### 2.3. Procedures

The quantitative results of this study were obtained through the application of a questionnaire before and after the implementation of the proposed activities (pre- and post-test). The questionnaire was pre-validated with a sample of 10 subjects. The time between the implementation of the activities and the application of the post-test was 3 months. Group I of the questionnaire included five questions; the first three questions included personal data (age, grade and school name), and the remaining questions evaluated the students’ knowledge of autochthonous plants and their identification. Group II of the questionnaire included 10 questions related to the definition of plant extracts and examples, the properties of the extracts, knowledge of microorganisms and their pathogenicity, and knowledge of scientific experiments/new medicine development. The questionnaire was administered during a regular class with a time limit of 45 min. Student participation was anonymous and voluntary. In the second phase of this project, the students who had completed the pre-test participated in the following sequence of activities.

### 2.4. Activity 0—Kick-Off: Creation of a Logo and Web Page (Facebook)

The objective of this activity was to create a logo and a web page to publicize this research activity. The web page can be found at (https://www.facebook.com/pages/Atividade-antimicrobiana-antitumoral-e-anti-inflamat%C3%B3ria-de-plantas/685696234827655). At this stage, we aimed at transmitting the importance of creating a brand as a strategy to promote/market a product or activity. We observed the students’ skills of entrepreneurship in terms of design/dissemination. It was our intention to show the importance of product presentation to arouse the consumer’s attention and desire.

### 2.5. Activity 1—Diagnose: Implementation of a Pre-Test

Initially, a questionnaire was applied to assess the students’ knowledge about the themes explored in the course of the study (Table 2).

### 2.6. Activity 2—Exploration of the Existing Scientific Gardens

The specific objective of this activity was to explore the three scientific gardens (100 m^2^ each) existing in the School D. Maria II, previously implemented through the Project “*Jardins com(s) Ciência*” (Gardens with science). These gardens are representative of three distinct climatic environments of Portugal, such as the Atlantic, the Mediterranean Mountains and the Mediterranean Lowlands. In the Atlantic Garden, the species are *Quercus robur*, *Acer pseudoplatanus*, *Betula alba*, *Crataegus monogyna*, and *Corylus avellana*; in the Mediterranean Mountains Garden, *Quercus pyrenaica*, *Quercus faginea*, *Erica australis*, *Cistus populifolius*, and *Rosa canina*; and in the Mediterranean Lowlands Garden, *Quercus suber*, *Quercus rotundifolia*, *Lavandula pedunculata*, *H. stoechos* and *Arbutus unedo*.

### 2.7. Activity 3—To Investigate/Discover

To study the antimicrobial, antitumor and anti-inflammatory activities of the plant extracts, the following species were selected from the Scientific Gardens: *C. populifolius*, *C. monogyna*, *E. australis*, *H. stoechas*, *L. pedunculata* and *R. canina*. Under the guidance of Professor Alberto Dias (AD, University of Minho-UM), the students prepared water and ethanol extracts of these plants and determined their phytochemical composition by high liquid pressure chromatography (HPLC-DAD-MS). Subsequently, the antimicrobial, antitumor and anti-inflammatory activities of these extracts were evaluated. This activity was held at the School of Health Sciences (UM) under the guidance of Professors Maria Manuel Azevedo (MMA), Fátima Baltazar (FB) and Filipa Ribeiro (FR).

The antimicrobial activity of extracts was tested in bacterial and fungal strains, including *E. coli*, *S. aureus*, *S. epidermidis* and *C. albicans*. At the beginning of this session, students were warned about the safety issues concerning work in a microbiology laboratory, including appropriate procedures, methodologies in handling microbial cultures, the type of biological safety cabinets used in microbiology laboratories (Class I, II and III) and basic rules for handling laboratory animals. However, as a safety measure, the students only handled non-pathogenic strains, namely *Saccharomyces cerevisiae*, *Lactobacillus casei* and *Schizosaccharomyces pombe*. The pathogenic strains above identified were only handled by the researchers involved in the activity.

Antitumor activity was assessed in two models of solid tumors, breast cancer (lines Hs578T, MDA-MB-231) and prostate (DU145, PC3). Anti-inflammatory activity was tested in a pre-clinical model of osteoarthritis. All of these activities were recorded in the form of photos/movies.

### 2.8. Activity 4—Application

The objective of this activity was to select the best extracts in terms of antimicrobial and anti-inflammatory properties, aiming to incorporate them in glycerin to make soaps and creams. This activity was performed by the students and was held at the D. Maria II School under the guidance of AD.

### 2.9. Activity 5—Listening to Science

The specific objective of this activity was to create a direct contact between students and researchers from the areas addressed in the project. This activity included a lecture performed by three specialists, followed by an active discussion (lasting about 1.5 h).

The lecture “Antimicrobial, antitumor and anti-inflammatory activity of autochthonous plants” was conducted by FB, FR and AD from the Universi ty of Minho. Initially, AD focused his intervention on the history of science emphasizing the use of plant extracts, revisiting the civilizations of Mesopotamia and Egypt, the Middle Ages, the Renaissance, up to the present. Subsequently, he presented several examples of the applicability of ointments/creams made from plant extracts with preventive/curative purposes. FB focused on the antimicrobial and antitumor properties of plant extracts, referring to examples in the literature that demonstrate their use in combating infectious diseases and cancer. Finally, FR commented on the anti-inflammatory properties of plant extracts, referring to examples that demonstrate their use beginning in ancient times to treat inflammatory diseases. This lecture was supported by a *Powerpoint* presentation and was finalized by presentation of a film illustrating the various steps of the project. The preliminary results of the antimicrobial, antitumor and anti-inflammatory activities were shown to the students at this point, highlighting their potential but also the need to repeat the experiments and use additional controls to confirm the results.

### 2.10. Activity 6—Communication Strategies

Communication of the activities developed in this project was promoted through a project web page on Facebook, the web page of the D. Maria II School, Local and Regional Newspapers, an Education Newsletter of V.N. Famalicão and the websites of the Schools of Science and Health Sciences (UM).

### 2.11. Activity 7—Lecture for the Educational Community

During the last week of classes, the students involved in the project organized a lecture under the supervision of MMA for the educational community. The aim was to present the various stages of the project and the results obtained including the soaps and ointments that were produced.

### 2.12. Activity 8—Evaluation: Implementation of a Post-Test

At the end of this study, a post-test was applied to investigate the students’ progress concerning their knowledge of the themes explored throughout the course of this study.

### 2.13. Data Collection

To obtain a broader and more inclusive representation of the project effectiveness, several methodologies were used, such as pre- and post-test analysis, observation of the students’ involvement in the tasks, assessment of activity reports, assessment of students’ feedback (Figure 1). All of the data collected in the pre- and post-test were processed and analyzed anonymously.

### 2.14. Data Analysis

Data obtained in the pre- and post-test were analyzed using SPSS software for Windows, version 18.0 (SPSS Inc., Chicago, IL, USA). Associations between variables were tested with Pearson’s Chi-square (χ^2^) tests with significance set at *p* < 0.05.

## 3. Results

### 3.1. Performance of the Students in the Pre- and Post-Test

In the group I questions, the data collected showed an overall improvement of the participants’ understanding of the concepts of autochthonous plants their features and identification (Table 2 and Table 3). In particular, there were significant differences for question Q5e. For the group II questions, there was a general improvement of the participants’ understanding of most of the concepts (Table 3). In particular, there were significant differences for questions, Q6, Q7, Q8a,c, Q9a,c,d, Q10b,d, Q12, Q13, Q14 and Q15. Regarding the control group, no significant improvements were detected, with the exception of question Q9c. Overall, there were significant improvements in the quality of the participants’ responses, as demonstrated by the enhanced scores for the questions presented.

### 3.2. Observation of the Students

**Figure 2:**
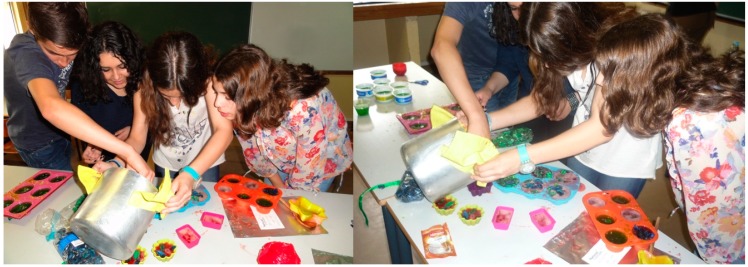
Preparation of soaps containing plant extracts by the students.

During activities 2 (Exploration of the existing scientific gardens), 3 (Investigate/Discover) and 4 (Application), teachers and researchers guided students by asking questions and pointing out interesting features concerning: (a) plants and their properties; (b) pathogenic microorganisms; (c) the history of science; and (d) scientific methodology. These types of approaches encouraged students to reflect on what they saw and experienced. It is noteworthy that all of the students participated very actively in the field/laboratory activities. It was also our feeling that they performed all of the tasks with pleasure, demonstrating interest and motivation for the activities (Figure 2).

### 3.3. Reports on Students’ Activities

With the help of the teachers, students reported the activities performed in the format of news that was published in regional newspapers. The first news article consisted of the presentation of the project, the second was related to the preparation of the plant extracts, the third focused on the antimicrobial, antitumor and anti-inflammatory properties of the plant extracts, the fourth reported the lecture conducted by AD, FB and FR and the last article was associated with activity 4 “Application” (see the Facebook page of the project).

### 3.4. Student’s Feedback

No relevant difficulties were identified during the execution of the described activities. Most students knew that plants may produce substances with pharmacological interest; however, they did not know that the plant extracts could be used to inhibit the growth of bacteria/fungi or cancer cells.

Most students stated that they were unfamiliar with the laboratory activities carried out during the study and frequently asked questions about the laboratory equipment, materials, and protocols. All of them were very careful in handling all laboratory materials. Their practical competencies and confidence improved considerably throughout the project and the participants were actively engaged in every task. The relevance of antibiotic resistance was emphasized several times by professor FB, and students revealed that they recognized the importance of this problem and even mentioned that they were going to share the information with friends and family.

At the end of the project, students were asked to write comments about the activities. Here, we transcribe a few: “It was a unique, interesting and fun experience”; “It was an experience that combined science and education and undoubtedly aroused the scientific curiosity of the youngsters”; “We worked as real scientists”; “It was a great experience, I learned a lot”; “I realized that mice may be happier in the laboratory than on the street”; “Thanks to animal experiments it is possible to find solutions for some diseases”; “I learned to work in a sterile environment and to use several materials in the laboratory”. Based on the student’s comments, we can conclude that they enjoyed participating in the project, they thought that the content was very interesting and up to date. Also, the promoters of the activities consistently felt that the environment was friendly and appealing.

### 3.5. Participants’ Engagement

**Figure 3:**
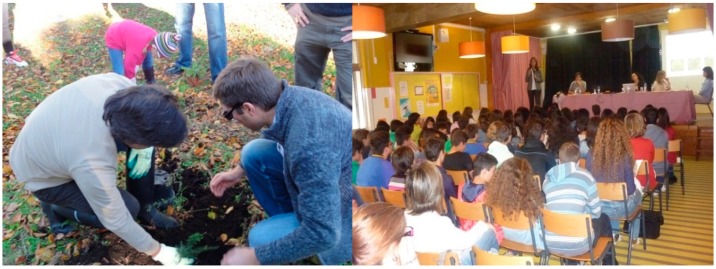
Exploitation of the “scientific gardens” and “scientific lectures”, with the participation of several members from the educational community.

In this study we created a collaborative teaching/learning process between teachers/researchers/students. All of the players were involved and participated dynamically in information exchange and in creative discussions, each sharing their own knowledge and experience. The value of the teacher’s knowledge in conducting education programs was evident, and the technical knowledge and enthusiasm of the researchers involved was crucial. This program was successful in promoting collaboration among all partners (Figure 3).

**Table 1:** Characteristics of the study population.

Students’ Groups	Grade	Number of Students	Age (Years)
Control Group (School Arnoso)	8th	19	12–16
Study group (School D. Maria II)	8th	19	13–15
Total	8th	38	12–16

**Table 2:** Questions used in the pre/post-test to investigate students’ progress in learning the themes developed in this project.

Question number	Group I Questions
Q1–3	Age, grade, school name
Q4	Synonyms of autochthonous plants
Q5	Identification of Portuguese autochthonous plants
	**Group II Questions**
Q6	Definition of plant extracts
Q7	Examples of plant extracts
Q8	Identification of microorganisms
Q9	Identification of pathogenic microorganisms
Q10	Medical applications of plant extracts
Q11	Knowledge about scientific experiments
Q12	Role of a control group in a scientific experiment
Q13	Definition of cell culture
Q14	Definition of experimental disease model
Q15	Knowledge on how the discovery of new medicines

**Table 3:** Comparison of the correct answers between the pre- and post-test (control and study groups) to the questions described in Table 2.

Questions	Study Group (*n* = 19)			Control Group (*n* = 19)		
	Pre-Test (%)	Post-Test (%)	*p* Value	Pre-Test (%)	Post-Test (%)	*p* Value
**Group I questions**						
**Q4** Identify the option which corresponds to synonyms of autochthonous plants (1 correct) a) Spontaneous, autochthonous, indigenous b) Exotic, autochthonous, introduced c) Autochthonous, native, introduced d) Native, exotic, autochthonous	26	47	0.179	26	0	**0.046**
**Q5** Identify the Portuguese autochthonous plants	1111	42	0.151	21	16	1.0
a) *Cistus populifolius*	4	32	0.090	16	16	1.0
b) *Crataegus monogyna*	21	37	0.732	37	58	0.194
c) *Erica australis*	11	21	1.0	21	11	0.660
d) *Helichysum stoechas*	0	74	**<0.001**	21	16	1.0
e) *Lavandula pedunculata*	50	68	0.721	68	37	0.051
f) *Rosa canina*	3	0		47	21	
Not answered						
**Group II questions**						
**Q6** Select the synonymous of plant extracts (1 correct) a) Dehydrated herbsb b) Plant water suspensionsc c) Plant partse d) Alcoholic herb solution	5	74	**<0.001**	11	5	1.0
**Q7** Give 2 examples of plant extracts (open question)	0	37	**0.008**	0	0	1.0
**Q8** Classify the microorganisms (bacteria, yeasts, filamentous fungi, …)	19	63	**0.020**	0	11	0.486
a) *Escherichia coli*	19	32	0.714	16	16	1.0
b) *Staphylococcus aureus*	34	74	**0.022**	16	16	1.0
c) *Staphylococcus epidermidis*	14	32	0.447	32	53	0.189
d) *Candida albicans*	14	0		37	32	
Not answered						
**Q9** Identify the pathogenic microorganisms	21	58	**0.045**	11	16	1.0
a) *Escherichia coli*	32	53	0.189	21	21	1.0
b) *Staphylococcus aureus*	26	74	**0.004**	11	47	**0.029**
c) *Staphylococcus epidermidis*	3	58	**0.001**	18	11	1.0
d) *Candida albicans*						
**Q10** Select what applies of plant extracts	95	100	1.0	95	79	0.34
a) Manufacture of medicines	26	84	**0.001**	53	47	0.746
b) Antimicrobial properties	74	90	0.405	74	79	1.0
c) Anti-inflammatory properties	37	79	**0.020**	26	32	0.721
d) Anti-tumor properties						
**Q11** Define a scientific experiment in your own words	79	100	0.105	84	74	0.693
**Q12** Identify the role of a control group in a scientific experiment (open question)	0	37	**0.008**	0	0	1.0
**Q13** Define cell culture (open question)	0	74	**<0.001**	0	0	1.0
**Q14** Define experimental disease model (open question)	0	63	**<0.001**	0	0	1.0
**Q15** Describe how new medicines can be discovered (open question)	5	79	**<0.001**	0	0	1.0

## 4. Discussion

Developments in fundamental science are essential for all societies; however, applied science today is also of crucial importance because it allows for the translation of science into economic development. For the European Commission, this concern is real and has been fostered by funding collaboration projects between Companies/Universities (FP7-SME). This position is highlighted in the new Community Framework-Horizon 2020 in which the European Commission intends to transfer knowledge to convert science into a product with added value. This transfer of know-how aims to create more competitive economies that meet the challenges they face. On the other hand, and concerning young people, the need to increase scientific literacy has been extensively recognized by the Portuguese Ministry of Education, which has implemented explicit guidelines to consolidate the teaching/learning process: “National Curriculum for Basic Education-Essential Skills” [26]. A study conducted with 2075 Portuguese students, spread across different school years, showed that only 37% of the respondents displayed an interest for science-related fields and that this interest was proportional to the degree of proximity with scientific areas [27].

To increase scientific literacy associated with innovation/application, we designed a project entitled “Antimicrobial, antitumor and anti-inflammatory activity of autochthonous plants and its valuation into real products: A study with Portuguese students”. The project provided an overview from the ecosystem to the species and aimed at creating a novel model of scientific and entrepreneurial culture able to stimulate proactive attitude in students. It was a hands-on approach built to enhance the participants’ understanding about autochthonous plants and the medical properties of plant extracts such as antimicrobial, antitumor and anti-inflammatory properties.

We used a convenience sample of students to allow rapid data collection in a short amount of time with limited resources. Information on the socio-economic and intellectual level of the participants was not collected. The data collected reveals that this project provided the students with a more intricate picture of autochthonous plants, plant extract medicinal properties, scientific experiments and experimental disease models by exposing them to accurate concepts and increasing their awareness about the importance of the protection of autochthonous plants. In the pre-test, the students demonstrated a lack of knowledge of autochthonous plants, and in the post-test, there was a significant increase in the number of correct answers. The results obtained in the pre-test are in accordance with “The Gallup Organization, 2010” report [28]. This study revealed that only 1% of the questioned Portuguese population considered invasive species to be the most important threat to biodiversity. Concerning plant identification, the low ability of the participants to identity plants observed in the pre-test is not unique with this age group because student knowledge of plant identification is also low in other countries [29]. Unfortunately, in the post-test, students still show an overall lack of knowledge on the subject, hinting at the need to further develop activities where their skills are improved.

Regarding the questions related to plant extracts, we observed a significant increase in the number of correct answers between the pre- and post-test. Similarly, with regard to the knowledge of microorganisms and their pathogenicity, a significant increase in the number of correct answers was detected. With respect to scientific experiments and new medicines, the results obtained in the post-test were quite satisfactory, with the biggest improvement obtained for questions related to experimental disease models. This improvement may be due to the impact of the emphasis given by the researchers to this matter during “Activity 3- Investigate/Discover” and Lecture 1.

An important part of this work was the comparison with the control group. In the whole, there was no significant improvement in the results from the pre- to post-test, with the exception of one question, which supports the positive results obtained with the study group.

Overall, we can conclude that the practical activities implemented herein were adapted to the target students, making it very easy to engage the students in understanding the concepts and problems proposed. The innovative practical activities, together with the lectures, complemented each other and allowed students to acquire a step-by-step understanding of the thematics explored. The activity “Application” was of crucial importance because the know-how acquired was applied to the creation of promising and innovative antimicrobial, antitumor and anti-inflammatory products, such as soaps and ointments. Furthermore, data from the literature revealed that teaching in the laboratory could facilitate the development of analytical and cognitive skills, such as the analysis of task requirements, questioning, and critically interpreting data. In addition, the learning of scientific topics improves thanks to a better understanding on how to apply certain theoretical concepts [30] and allowing students to reorganize and build their know-how and skills [31]. With the introduction of laboratory work, students should therefore always have the chance to manipulate experimental materials and thus gain hands-on experience [32]. A recent work with students from the basic school level provided a novel perspective for the study of the human body by using a hands-on approach, with self-perceived advantages [33]. The American National Science Teachers’ Association agrees that laboratory experience is “so integral to the nature of science that it must be included in every science program for every student” because experimental activities help to develop a wide variety of organizational, creative and communicative skills. This association recommends for middle-level schools (ages 11–13) that a minimum of 80% of science instruction time should be spent on laboratory-related practices [34]. Contextualized practical activities are known to promote the ability to connect observable and conceptual dimensions [35].

Another important objective of this project was the creation of innovative products. The teachers/researchers involved in this project were also very excited and consider the activities adapted to the students age and curricula. The preliminary experimental results obtained in study of the antimicrobial, antitumor and anti-inflammatory properties of plants in Activity 3 were considered by the researchers of the University of Minho to be very interesting and promising, although additional experiments should be performed and new controls introduced prior to the publication of the results.

This project represents a contribution to enhance awareness of the need to protect autochthonous plants and value their potential for the human health from both an ecological and economic perspective. This type of activities with students will help prepare future generations of citizens who have a more active and positive attitude towards sustainable development.

The post-test was administered 3 months after the completion of the experimental activities; however, it would be important to re-apply it after a longer period of time to infer on the long-term retention of knowledge.

To efficiently advertise this project and promote knowledge transfer, we used various tools, such as a Facebook pages, official internet sites for the school and the University of Minho, local and regional newspapers and lectures for the entire school community.

The activities developed in this project promoted discussion, and the debate allowed students to better understand the message underlying the activities. One of the key roles of the researchers involved in this work was to help students make connections between what they are seeing and its meaning, with scientific validation. The students expressed their satisfaction about the way in which practice and theory were integrated, they performed all of the tasks with pleasure, demonstrating a great interest and motivation. They also appreciated the chance to perform experimental work, which is not common practice in their own schools. According to the student’s comments, we can conclude that they valued participating in the project.

## 5. Conclusions

In summary, this project shows that there are relevant educational benefits attained when incorporating hands-on activities in science education programs and could encourage other teachers to promote similar activities. The main objectives proposed in the present study were achieved and our research questions were answered.
